# Improved Drought Stress Response in Alfalfa Plants Nodulated by an IAA Over-producing *Rhizobium* Strain

**DOI:** 10.3389/fmicb.2017.02466

**Published:** 2017-12-14

**Authors:** Roberto Defez, Anna Andreozzi, Michael Dickinson, Adrian Charlton, Luca Tadini, Paolo Pesaresi, Carmen Bianco

**Affiliations:** ^1^Institute of Biosciences and BioResources – National Research Council, Naples, Italy; ^2^Fera Science Ltd., National Agri-Food Innovation Campus, Sand Hutton, United Kingdom; ^3^Dipartimento di Bioscienze, Università degli Studi di Milano, Milan, Italy; ^4^Dipartimento di Scienze Agrarie e Forestali, Università degli Studi di Milano, Milan, Italy

**Keywords:** rhizobium, legume, nitrogen fixation, drought stress, indole-3-acetic acid, ABA, ethylene, photosynthesis

## Abstract

The drought–stress response in plant involves the cross-talk between abscisic acid (ABA) and other phytohormones, such as jasmonates and ethylene. The auxin indole-3-acetic acid (IAA) plays an integral part in plant adaptation to drought stress. Investigation was made to see how the main auxin IAA interacted with other plant hormones under water stress, applied through two different growth conditions (solid and hydroponic). *Medicago sativa* plants nodulated by the *Ensifer meliloti* wild type 1021 (*Ms*-1021) and its IAA-overproducing RD64 derivative strains (Ms-RD64) were subjected to drought stress, comparing their response. When the expression of *nifH* gene and the activity of the nitrogenase enzyme were measured after stress treatments, *Ms-RD64* plants recorded a significantly weaker damage. These results were correlated with a lower biomass reduction, and a higher Rubisco protein level measured for the *Ms-RD64*-stressed plants as compared to the *Ms-1021*-stressed ones. It has been verified that the stress response observed for *Ms-RD64*-stressed plants was related to the production of greater amount of low-molecular-weight osmolytes, such as proline and pinitol, measured in these plants. For the *Ms-RD64* plants the immunoblotting analysis of thylakoid membrane proteins showed that some of the photosystem proteins increased after the stress. An increased non-photochemical quenching after the stress was also observed for these plants. The reduced wilting signs observed for these plants were also connected to the significant down-regulation of the *MtAA03* gene involved in the ABA biosynthesis, and with the unchanged expression of the two genes (*Mt-2g006330* and *Mt-8g095330*) of ABA signaling. When the expression level of the ethylene-signaling genes was evaluated by qPCR analysis no significant alteration of the key positive regulators was recorded for *Ms-RD64*-stressed plants. Coherently, these plants accumulated 40% less ethylene as compared to *Ms-1021*-stressed ones. The results presented herein indicate that the variations in endogenous IAA levels, triggered by the overproduction of rhizobial IAA inside root nodules, positively affected drought stress response in nodulated alfalfa plants.

## Introduction

Environmental factors imposing stress on plants, including drought, salinity, heat, chilling, freezing, etc. cause widespread crop losses throughout the world. In particular, the constant increase in temperatures and the water scarcity are increasing the frequency of severe drought conditions. Drought stress reduces leaf size, stem extension and root proliferation, thus reducing water-use efficiency. CO_2_ assimilation by leaves is hampered mainly by stomatal closure, membrane damage and altered functioning of enzymes involved in this process (Flexas and Medrano, 2002). Enhanced metabolite flux through the photorespiratory pathway generates reactive oxygen species, leading to increased oxidative load on the tissues, thus severely damaging the biological macromolecules (Zhu, 2002; Osakable et al., 2014).

So far, different strategies have been developed to improve water adsorption and stress tolerance in crop plants, including legumes. Clarification of the mechanisms controlling the perception and the transduction of stress signals in these plants is essential. Environmental stresses require plants to perceive and react to these signals in a highly coordinated and interactive manner. Stress conditions greatly affect gene expression, leading to modifications in the composition of plant proteome and metabolome (Urano et al., 2010; Goldack et al., 2014; Zhang et al., 2014; Wang et al., 2016).

It is widely recognized that the perception of abiotic stresses triggers the interaction of the signal transduction cascades, with the pathways transduced by phytohormones. The fluctuation of stress-responsive hormones plays a central and coordinate role in the regulation of growth response under stress conditions (Archarya and Assman, 2009; Wilkinson and Davies, 2010; Harrison, 2012; Du et al., 2013).

Changes in gene expression induced by drought stress overlapping with hormone-regulated gene expression have been reported in many studies in which the turgor loss, recognized as a major physiological determinant of plant water stress response, has been induced by both dehydration or increasing extracellular solute concentration (Huang et al., 2008; Li et al., 2016). During drought stress, abscisic acid (ABA) is the primary phytohormone that triggers short-term responses, such as stomatal closure, and long-term responses, favoring maintenance of root growth optimizing water uptake (Sreenivasulu et al., 2012; Daszkowska-Golec and Szarejko, 2013; Sah et al., 2016). Several reports show that stomatal function is also dependent on other hormones (auxin, cytokinin, ethylene, brassinosteroids, jasmonates, and salicylic acid) and on their mutual interaction. In addition, all these hormones are involved in the regulation of stress-related genes (Seki et al., 2003; Öktem et al., 2008; Mickelbart et al., 2015). Many strategies have been used to improve abiotic stress tolerance in crop plants, and phytohormones engineering could be an attractive strategy to improve the efficiency of water use (Hu and Xiong, 2014; Wani et al., 2016). Over-expression of genes involved in ABA biosynthesis/catabolism has led to increased drought tolerance, but in some cases impaired growth, due to pleiotropic effects, has also been observed (Cutler et al., 2010; Park et al., 2015). The production of transgenic poplar plants, producing higher levels of auxin and exhibiting an increased abiotic stress tolerance (Ke et al., 2015), has recently been reported. In these transgenic plants one member of the *YUCCA* genes (*YUCCA6*), playing an important role in maintaining auxin levels in *Arabidopsis* and many other plants, such as tomato, maize, rice and petunia, was expressed under the control of an oxidative stress-inducible promoter. Increased free indole-3-acetic acid (IAA) levels and improved drought stress tolerance connected with reduced levels of reactive oxygen species and delayed leaf senescence have been observed for these plants (Ke et al., 2015). High-auxin phenotypes, such as rapid shoot growth, retarded main root development with increased root hair formation, and enhanced drought tolerance based on reduced water loss had also been observed in potato plants overexpressing the *Arabidopsis YUCCA6* gene (Kim et al., 2013). This study has hypothesized that the auxin overproduction could lead to adjustments in the synthesis or distribution of other hormones, such as ABA, known to control water balance through its effect on stomata and the expression of osmotic-stress-tolerance genes. The production of plant varieties with improved stress resistance could speed up by unraveling the mechanisms underlying the correlation of auxin biosynthesis with environmental cues.

The endogenous pool of IAA in plants may be altered by the acquisition of IAA secreted by soil bacteria, which in the case of legumes are mainly the rhizobia. Within the nodules on the legumes roots, the IAA produced by rhizobia could alter the endogenous plant IAA, resulting in plant growth promotion or inhibition (Mathesius et al., 1998; Bianco et al., 2014; Ansari et al., 2017).

It has been previously verified that the inoculation of *Medicago* plants with the RD64 *Ensifer meliloti* strain, a derivative of the *Ensifer meliloti* 1021 engineered to overproduce the auxin IAA (Defez and Spena, 1998; Pandolfini et al., 2000), led to the stimulation of the N and C metabolism and to the improvement of N-fixation, salt tolerance, mineral phosphate solubilization, and plant yield (Bianco and Defez, 2009, 2010a,b; Bianco et al., 2009; Imperlini et al., 2009). These effects were connected to the higher IAA concentration, measured in the nodules deriving from plants infected with the RD64 strain as compared to those infected with the control strain. The induction of both the *nifH* gene expression and the nitrogenase enzyme activity has also been observed in diazotrophic bacteria associated with cereals in response to increased IAA availability (Defez et al., 2017), thus speculating that the activation of nitrogen fixation triggered by IAA is a widespread effect. The different phenotypes observed for plants infected with 1021 and RD64 strains, even under stress conditions, were thought to be consistent with alterations in the biosynthesis or accumulation of other important hormones controlling plant growth as result of the auxin accumulation at root level.

The main purpose of this work was to validate this hypothesis and to analyze the response of *Medicago* plants nodulated by the IAA-overproducing RD64 and the wild-type 1021 strains to water stress imposed through water deprivation and polyethylene glycol (PEG)-treatment.

The ABA-response to drought and the main mechanisms involved in the response of plants to abiotic stress were analyzed in *Ms*-RD64 plants over-expressing IAA within root nodules as compared to *Ms*-1021 ones. Rubisco large subunit amount, thylakoid proteins, and non-photochemical quenching (NPQ) were measured. Proline and pinitol content, as well as nitrogen-fixing activity were also tested for nodulated *Medicago* plants subjected to stress treatment. The expression of genes involved in ethylene signaling pathway and the amount of ethylene produced in *Ms*-RD64 plants were quantified.

The combination of phenotypic, molecular and biochemical approaches produces useful complementary data that can help clarify the crosstalk between phytohormones signaling under stressful environmental condition.

## Materials and Methods

### Bacterial Strains, Growth Conditions, and Plasmids

The bacterial strains used in this study are the *Ensifer meliloti* 1021 (Galibert et al., 2001) and its IAA-overproducing derivative, RD64, containing the p-*iaaMtms2* construct in which the coding region of the *iaaM* gene of *Pseudomonas savastanoi* was positioned downstream from an 85 bp promoter sequence, and the *tms2* coding region of *Agrobacterium tumefaciens* was placed at the 3′ end of *iaaM* gene, as described by Defez and Spena (1998) and Pandolfini et al. (2000). Bacterial strains were aerobically grown at 30 °C in TYR medium (Bianco and Defez, 2010a). Streptomycin (200 mg l^-1^ for *E. meliloti* 1021) and spectinomycin (200 mg l^-1^ for *E. meliloti* RD64) were included as required.

### Plant Material

*Medicago sativa* seeds were surface sterilized as reported by Bucciarelli et al. (2006). Sterilized seeds were germinated for three days and then transferred into hydroponic units as previously described by Bianco and Defez (2009) or into pots containing perlite-sand soil in 3:1 ratio. The inoculation of germinated seeds with bacteria was carried out by adding a bacterial suspension (10^4^ bacteria/ml) directly to the liquid medium (hydroponics growth) or to the roots of the growing plants (potted growth). The age of the plants was calculated from the day of inoculation. Drought stress was applied to 28-day-old plants under two different conditions: PEG-treatment and water withholding. Twenty one-day-old plants were only used for ethylene measurement as will be described in the specific paragraph. To induce controlled osmotic drought stress in nutrient solution the medium of the hydroponic units was replaced with a 15% PEG-6000 water solution for 28 h. Control plants were kept in regular hydroponic solution. To apply drought under perlite-sand soil growth conditions, alfalfa plants were not watered for 6 days, while control plants were daily watered. These treatment times were selected after evaluating different conditions allowing us to have still alive plants nodulated by both *E. meliloti* 1021 and RD64 strains. After 28-hour-PEG-treatment and 6-day-water-deprivation, one set of the plants from each treatment was directly subjected to non-destructive analysis, while another set was immediately collected, frozen in liquid nitrogen and stored at –80°C for further analyses.

Greenhouse conditions were those previously reported by Bianco and Defez (2009). Bacteria (*Ensifer meliloti* 1021 and RD64) were isolated from nodules deriving from 10-day-old plants, and their identities verified by their antibiotic resistance patterns.

### qRT-PCR Analysis

Roots and shoots of alfalfa plants subjected to both water deprivation and PEG-treatment as above described were used for the isolation of RNA as previously reported (Bianco et al., 2014). Residual DNA present in the RNA preparations was removed by using the RNAse-free TURBO DNase I Kit (Applied Biosystems) according to the manufacturer’s instructions.

After purification and quality checking by agarose gel electrophoresis, the RNA concentration was determined by absorbance at 260 nm and the RNA was stored at –20°C until further use. First-strand cDNA was synthesized from 1 μg of total RNA with the RETROscript kit (Applied Biosystems) and random decamers, according to the manufacturer’s instructions. qRT-PCR was performed as previously described (Bianco and Defez, 2009).

Specific primer pairs for *MtP5CS2*, *ETR1*, *CTR1*, and *EIN2* genes were those reported in Bianco and Defez (2009). Specific primer pairs for *nifH* gene were those reported in Defez et al. (2016). Specific primer pairs for *MtAAO3*, *Mt-2g006330*, and *Mt-8g095330* genes, designed using Primer3 software, were as follows: for *MtAAO3*, 5′-AAACTCGGTTGTGGTGAAGG-3′ and 5′-TGGAATCCTGCAAACCTTTC-3′; for *Mt-2g006330*, 5′- GGAGCATACCCATTTGAGGA -3′ and 5′- GCTGGACTTGCCACAAAAAT -3′; for *Mt*-8g095330, 5′- GTGTGGCTAGGCTTTTGAGG -3′ and 5′- GGTCAAAACCACCTCCTTGA -3′.

Primers for *Actin* and *Mtc27* were included in all the qRT-PCR analyses for the purpose of data normalization. During the reactions, the fluorescence signal due to SYBR Green intercalation was monitored to quantify the double-stranded DNA product formed in each PCR cycle. Results were recorded as relative gene expression changes after normalizing for *Actin* and *Mtc27* gene expression and computed using the comparative CT method (2^-ΔΔCT^) as described in Livak and Schmittgen (2001).

The 2^-ΔΔCT^ value was >1 for genes more highly expressed in stressed plants and <1 for genes more highly expressed in control plants.

qRT-PCR data are the mean ± SD of at least four biological replicates.

### Measurement of Ethylene Production

For ethylene analysis, plants grown in pots containing perlite-sand soil were sampled at 21 days after inoculation and transferred whole into a 20 mL glass vials containing 4 mL of 20% (w/v) PEG-6000, avoiding damage caused by the sheathing of the stem. Control plants were transferred into vials containing regular hydroponic solution. The vials were sealed with a silicon lid and incubated for 24 h in the dark at 21°C. Using a gastight syringe 1.0 ml sample was then taken through a silicon stopper and the amount of ethylene produced was determined by a gas chromatograph (GC) equipped with a flame ionization detector as described by Defez et al. (2017). The ethylene quantification was normalized to the fresh weight of whole plants measured after the PEG-treatment.

### Chlorophyll a Fluorescence and NPQ Measurements

*In vivo* Chlorophyll a fluorescence was measured for *Medicago* plants grown in hydroponic conditions and subjected to PEG-treatment as above described using the Dual-PAM-100^1^ (Walz) as previously described (Pesaresi et al., 2009). The maximum quantum yield of photosystem II (PSII), F_V_/F_M_ (*F*_m_–*F*_0_/*F*_m_), the effective quantum yield of PSII, Y(II), and the NPQ were quantified. Six plants of *Ms*-0121 and *Ms*-RD64 were analyzed and average values and standard deviations of *F*_V_/*F*_M_, Y(II), and NPQ were calculated.

### Acetylene Reduction Assays (ARA)

Alfalfa plants subjected to both water deprivation and PEG-treatment as above described were analyzed for acetylene reduction activity as reported in Camerini et al. (2008) with some modifications described in Defez et al. (2017). Activity data are the mean ± SD of at least four biological replicates.

### SDS–PAGE and Immunoblot Analysis

#### Rubisco

Immunoblot detection of Rubisco large subunit was carried out on alfalfa plants subjected to water deprivation as above described. The soluble leaf proteins were extracted in ice cold extraction buffer containing 100 mM Tri-HCl (pH 8.0), 10 mM MgCl_2_, 10 mM NaHCO_3_, 1 mM EDTA, 12.5 % (v/v) glycerol, 0.1% (v/v) Triton, 1% (w/v) insoluble polyvinyl polypyrrolidone, and the complete mini EDTA-free protease inhibitor cocktail (Sigma–Aldrich). The samples were homogenized using a Tissue Lyser (Qiagen) and the extract clarified by centrifugation at 13,000 × *g* for 30 min at 4°C. The content of soluble proteins was measured by the Bradford protein assay with the BSA as a standard. The supernatants were stored at -20°C prior to SDS-PAGE and immunoblot analysis. The proteins were separated by 12.5% SDS-PAGE with a Mini Protean II cell (BIO-RAD) according to Laemmli (1970). After electrophoresis, proteins were blotted onto PVDF membranes according to the standard procedures. Prestained SDS MW Standards (6,521-194-731 kDa, BIO-RAD) were used to control the effectiveness of the transfer. Blots were probed with anti-RbcL (Rubisco large subunit, Agrisera) and anti-rabbit IgG AP-conjugated (BIO-RAD) as secondary antibodies and developed with nitroblue tetrazolium and 5-bromo-4-chloro-3-indolyl phosphate. Developed immunoblot were scanned using VersaDoc imaging system (BIO-RAD) and processed using the Quantity One software (BIO-RAD). For immunoblot quantification RbcL protein standard (AGRISERA) was subjected to SDS-PAGE and immunoblot. Electrophoresis and immunoblot were repeated with four different biological replicates and one representative picture of the results is given.

#### Thylakoid Membrane Proteins

Four biological replicates of leaf samples deriving from four independent alfalfa control plants and those subjected to PEG-treatment were collected immediately after the Chlorophyll a measurement as above described and total protein extracts were prepared according to Martínez-García et al. (1999). Total proteins deriving from 2 mg of leaf fresh-weight were fractionated by SDS-PAGE (12% acrylamide [w/v]; (Schagger and von Jagow, 1987) and transferred to nitrocellulose membranes. Replicate filters were probed with antibodies specific for PSI (PsaD), PSII (PsbO), Cyt b6/f (PetA and PetC), ATPase (ATPase-β) subunits, PSI (Lhca2), and PSII (Lhcb4) antenna proteins obtained from Agrisera^2^. The antibodies specific for the PsbD protein (PSII subunit) was kindly provided by Roberto Barbato (University of Eastern Piedmont). The immunoblot signals were quantified by using the ImageJ software^3^. Electrophoresis and immunoblot were repeated with four different biological replicates and one representative picture of the results is given.

### Measurement of Metabolites

L-glutamic acid, L-proline, D-glucose, sucrose, D-pinitol, malic acid, and citric acid were all purchased from Sigma–Aldrich (Gillingham, United Kingdom). All had a purity >98%. Methanol, acetonitrile, and formic acid were all purchased from Fisher Scientific (Loughborough, United Kingdom).

Dried root and shoot of alfalfa plants subjected to PEG-treatment as above described were milled using a “Mini Beadbeater” ball mill (BioSpec, Bartlesville, OK, United States) for approximately 30 min to convert the sample into a fine homogenous powder. The ground sample (5 mg ± 0.05 mg) was accurately weighed into a 2 mL eppendorf tube before addition of 1 mL of extraction solvent (1:1 (v/v) methanol: water). Metabolites were extracted into the solvent by shaking for 30 min. The solid material was then removed by centrifugation at 8,765 *g* for 10 min and the supernatant diluted a further 400-fold with ultrapure water (18.2 Ω filtered using Milli-Q Reference Water Purification System, Millipore, Merck Group, London, United Kingdom) before analysis by Liquid Chromatography – High Resolution Mass Spectrometry (LC-HRMS).

Liquid Chromatography (LC) analysis was performed on an Accela High Speed LC system from Thermo Scientific (Waltham, MA, United States). The analytical column used was an ACE Excel AQ (Advanced Chromatography Technologies, United Kingdom) 150 mm × 3 mm, 100 Å. Mobile phase A (MPA) was 0.1% formic acid in HPLC water, mobile phase B (MPB) was 0.1% formic acid in acetonitrile. Linear gradient elution was applied over 10 min from 100% MPA to 100% MPB. The gradient was then held for 2 min at 100% B before re-equilibration with 100% A for a further 2 min. Chromatographic run times were approximately 15 min per injection. The LC flow rate was 0.4 ml min^-1^ and the column temperature was 25°C. Sample injection volume was 10 μl.

The MS used was an Exactive Orbitrap high resolution mass spectrometer from Thermo Scientific (Waltham, MA, United States) with a mass resolution of 50,000 at *m/z* 200 and the advanced gain control set to “Balanced”. Maximum injection time was 100 ms. Ionization was by heated electrospray (H-ESI) and responses were assessed in both positive and negative mode with sheath gas set to 60 and aux gas at 17 (arbitrary units). The capillary temperature was 250°C and heater temperature 300°C. Spray voltage was 3.5 Kv for positive ion acquisition and 3 Kv for negative acquisition. Quantification of each metabolite was undertaken using a matrix matched standard addition approach, adding four levels of analytical standard between 0.1 and 1 μg/ml, spiked into spare control material for both shoots and roots. Glutamic acid, proline, sucrose, and pinitol were determined in positive ion mode whereas glucose, malic acid, and citric acid were determined in negative ion mode. Excel (Microsoft Corporation, Washington, DC, United States) and Xcalibur software (Thermo Scientific Waltham, MA, United States) were used to identify and quantify each metabolite. Due to problems with the analytical standard pinitol was not quantifiable and only relative MS responses were evaluated.

### Data Analysis

When not specifically indicated results are presented as means ± standard deviation of at least four biological replicates. The significance of the data was confirmed by Student’s *t*-test.

## Results

### Drought Stress Physiology of *Medicago sativa* Plants

Integrating physiological, transcriptomic, and metabolomic measurements were carried out to analyze the effects of drought stress on *Medicago* plants nodulated by 1021 and RD64 strains, under perlite-sand soil (water withholding) and hydroponic conditions (PEG-treatment).

The experimental conditions used for the two treatments were those for which *Ms*-1021 and *Ms*-RD64 plants, though being still alive, showed different physiological responses.

**FIGURE 1:**
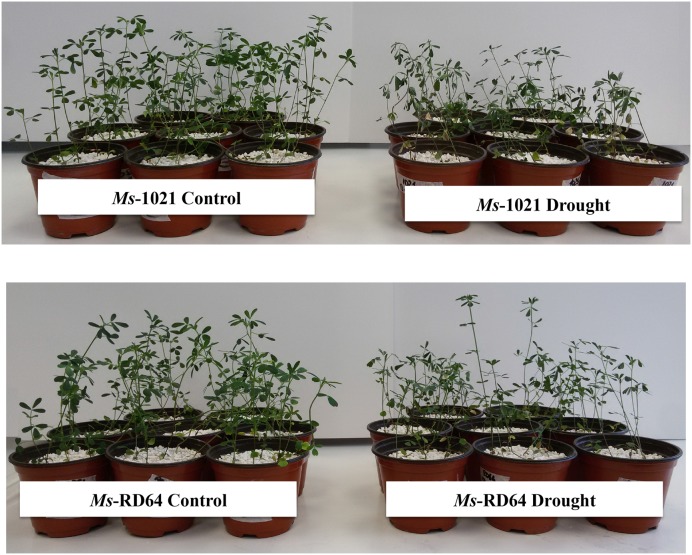
Phenotypes of nodulated *Medicago* plants subjected to drought stress. Four-week-old plants were subjected to drought stress by withholding watering for 6 days. Control plants were daily watered all the time. Photographs were taken at the end of the stress treatment.

After 6 days of no watering the *Ms*-1021 plants were almost completely wilted; by contrast, the *Ms*-RD64 plants were only partially wilted, maintaining their turgor as compared to their daily watered controls (**Figure 1**). Thus, meant that *Ms*-1021 plants were more affected by water deprivation than *Ms*-RD64 plants. A water deprivation applied beyond the 6th day led to the almost complete death of *Ms*-1021 plants (data not shown).

**FIGURE 2:**
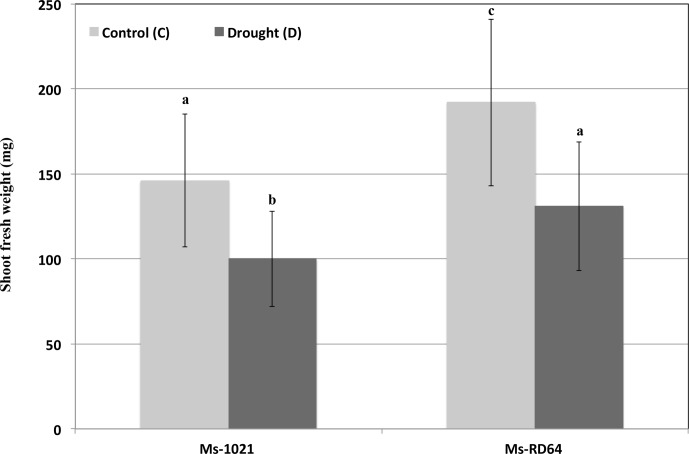
Effect of drought stress on nodulated *Medigaco sativa* growth. Plants nodulated by the wild type and the IAA-overproducing RD64 strains at 28 days after inoculation were subjected to drought conditions by withholding watering for 6 days. Data presented are the means ± SD (*n* = 25). Different letters are used to indicate means that differ significantly according to Tukey’s test (*P* < 0.01).

When the shoots fresh weights of nodulated *Medicago* plants were measured after six days of water deprivation a similar decline was recorded for both *Ms*-1021 and *Ms*-RD64 plants as compared to their controls (**Figure 2**). Nevertheless, the fresh weight data obtained for *Ms*-RD64-stressed plants were not statistically different from those registered for *Ms*-1021 non-stressed control plants.

**FIGURE 3:**
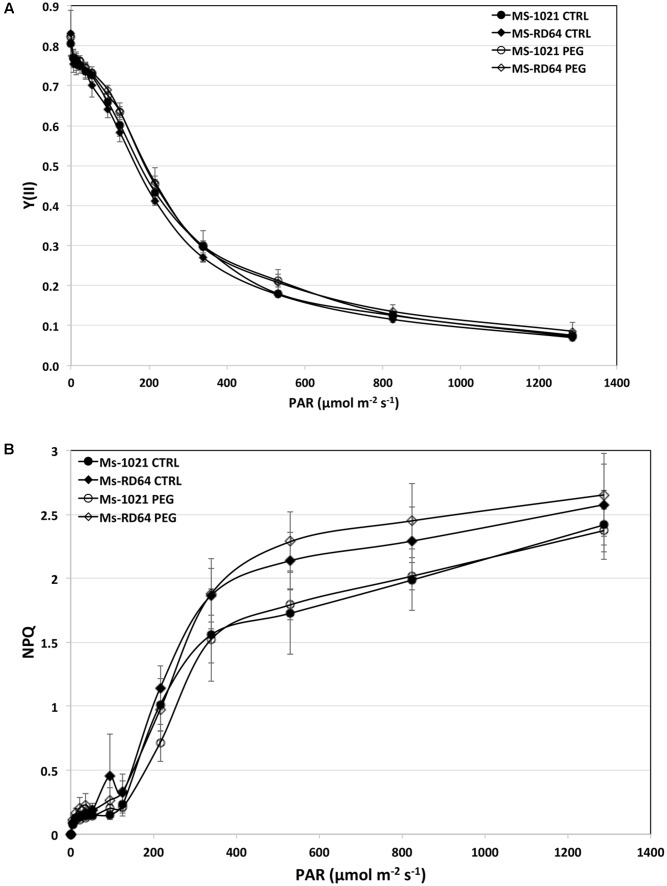
Light-dependent changes of the effective PSII quantum yield [Y(II)] **(A)** and non-photochemical quenching (NPQ) **(B)**. *Ms*-1021 and *Ms*-RD64 plants at 28 days after inoculation were treated for 28 h with 15% PEG-6000. At the end of PEG-treatment leaves from control (CTRL) and stressed (PEG) plants were immediately exposed for 15 min to different light intensities and then dark-adapted for 1 h at room temperature before measurements. Data presented are the mean ± SD of six biological replicates.

To provide insights into the *Medicago* ability to tolerate drought stress and the extent to which it damages the photosynthetic apparatus the chlorophyll a fluorescence and the NPQ were measured after 28 h of controlled osmotic drought stress (PEG-treatment). No significant difference in the PSII yield was observed under different light intensities for both control and stressed plants (**Figure 3A**). By contrast, a gradual and significant increase in NPQ value was registered for both control and stressed *Ms*-RD64 plants. Nevertheless, the increase was more evident for *Ms*-RD64-stressed plants up to 1287 μmol m^-2^ s^-1^ intensity (**Figure 3B**).

The symbiotic phenotype of nodulated *Medicago* plants was studied by measuring the activity of the nitrogenase enzyme through an acetylene reduction assay (ARA), as many of the proposed mechanisms responsible for decreasing activity in root nodules under water stress involve the nitrogenase (the enzyme responsible for N_2_ conversion to ammonia) as a key target. The data reported in **Table 1** show that both water deprivation and PEG-treatment had negative effects on N_2_ fixation of *Ms*-1021-stressed plants for which a nearly 40% decrease was recorded. However, a lesser activity decrease was measured for the *Ms*-RD64-stressed plants.

**Table 1:** Nitrogenase activity for nodulated *Medicago* plants subjected to drought stress.

Sample	Nitrogenase activity (nmol C_2_H_4_ plant^-1^ min^-1^)					
	Water deprivation^a^	Ratio	*P* value	PEG-treatment^b^	Ratio^c^	*P* value
*Ms*-1021 Control	1.8 ± 0.2			6.0 ± 1.0		
*Ms*-1021 Drought	0.8 ± 0.2	0.44	<0.002	3.4 ± 1.1	0.57	<0.02
*Ms*-RD64 Control	3.6 ± 1.0			8.7 ± 0.8		
*Ms*-RD64 Drought	2.0 ± 0.3	0.55	<0.03	7.5 ± 0.9	0.86	0.15

^a^*Half of the 28-day-old plants were subjected to drought conditions by withholding watering for 6 days, while another half were kept with daily watering until non-destructive analysis and harvesting. *^b^*To induce controlled osmotic drought stress in nutrient solution, half of the 28-day-old plants were transferred to the hydroponic water solution supplemented with 15% PEG-6000 for 28 h, while another half was kept in regular hydroponic solution until non-destructive analysis and harvesting. Data presented are the mean ± SD (*n* = 4). *^c^*Ratio calculation was based on control plants. The significance of the data was evaluated by Student’s *t*-test*.

### Rubisco Content and Thylakoids Membrane Proteins

**FIGURE 4:**
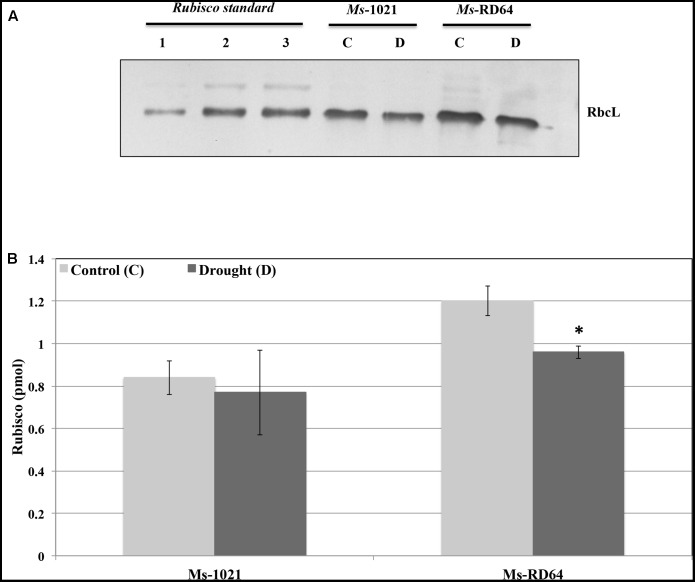
Immunoblot detection of Rubisco large subunit in extracts from *Medicago sativa* plants subjected to water deprivation. One microgram of total proteins were extracted from *Medicago* plants nodulated by the wild type and the IAA-overproducing RD64 strains and grown under control (C) and drought-stress (withholding watering for 6 days) (D) conditions. Protein extracts were separated by SDS-PAGE, transferred to nitro-cellulose membrane and probed with anti-RbcL antibodies (RbcL – Rubisco large subunit). **(A)** 0.3 pmol (lane 1), 0.6 pmol (lane 2), and 0.9 pmol (lane 3) of Rubisco protein standard loaded for the purpose of quantification. **(B)** Immunoblot quantification of RbcL protein. Data presented are the mean ± SD of four biological replicates. ^∗^*P* < 0.05, compared with the control plants (by Student’s *t*-test).

In the present study, immunoblot analysis performed using polyclonal antibodies against Rubisco large subunit (RbcL) showed that *Ms*-RD64 non-stressed plants contained more than 40% RbcL content as compared to *Ms*-1021 non-stressed plants. After six days of drought stress (**Figure 4**) reduced RbcL levels were measured. A slight decrease was observed for *Ms*-1021-stressed plants and was more evident for *Ms*-RD64-stressed ones. However, despite the decline, the RbcL level measured for the *Ms*-RD64-stressed plants was still 25% higher than that recorded for the *Ms*-1021 non-stressed plants.

**FIGURE 5:**
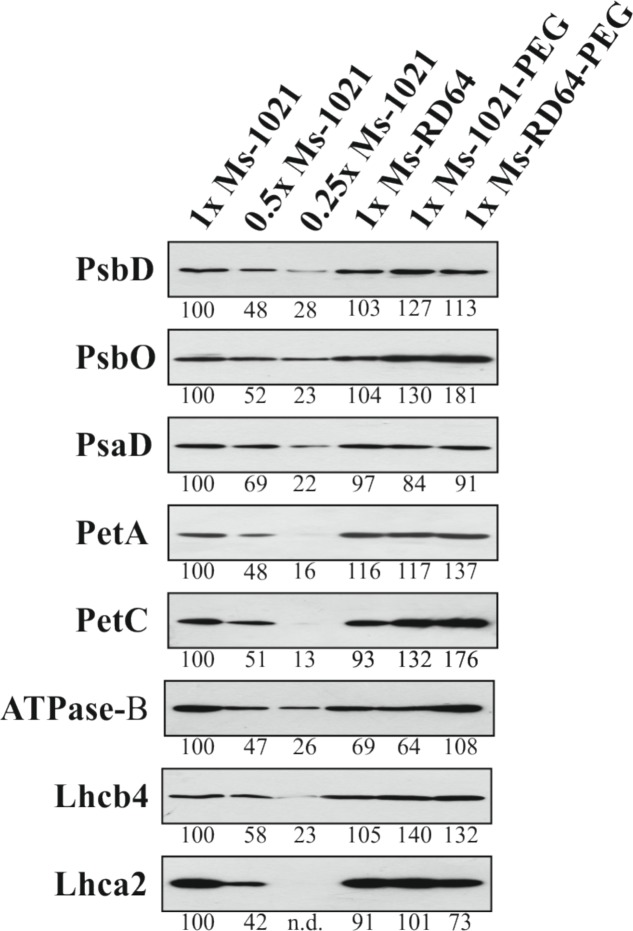
Immunoblot analysis of thylakoid proteins from *Medicago sativa* plants subjected to PEG-treatment. Total protein deriving from 2 mg of leaf fresh-weight of *Medicago sativa* plants nodulated by the wild type and the IAA-overproducing RD64 strains were fractionated by SDS–PAGE and transferred to nitrocellulose membranes. Replicate filters were probed with antibodies specific for PsbD (PSII), PsbO (PSII), PsaD (PSI), PetA (Cyt b6/f), PetC (Cyt b6/f), Lhcb4 (PSI), and Lhcb4 (PSII). The level of individual protein signals, quantified in controls and PEG-treated plants by ImageJ software (http://imagej.nih.gov/ij/index.html), is indicated below the bands. n.d., not detectable. Electrophoresis and immunoblot were repeated with four different biological replicates and one representative picture of the results is given.

The level of proteins related to PSI, PSII, the ATP synthase complex and cytochrome *b6/f* complex was investigated by immunoblot analysis to test the effect of drought stress on the four major multi-subunit protein complexes involved in the control of photosynthesis in plants in control and drought-stressed plants (**Figure 5**). The levels of PsbD, PsaD, PetA, and Lhca2 did not change significantly in drought-stressed plants compared to control ones. Significant increase was observed for PsbO, PetC and Lhcb4 proteins with the highest levels measured for the *Ms*-RD64 stressed plants. The level of Lhcb4 increased equally in the *Ms*-1021 and *Ms*-RD64 stressed plants. For ATPase-B protein the exposure to drought stress led to opposite results in the two stressed samples: a significant decrease in the protein level was measured for *Ms*-1021 stressed plants, whereas a 56% increase was observed for *Ms*-RD64 stressed plants.

### qRT-PCR Analysis

qRT-PCR analysis was performed to evaluate the effect of drought stress on the expression levels of selected genes involved in key pathways altered by water stress, such as the nitrogenase biosynthesis (*nifH)*, proline biosynthesis from glutamic acid (*MtP5CS2)*, ABA biosynthesis (*MtAAO3)* and signaling (*Mt-2g006330, Mt-8g095330*), and ethylene signaling (*ETR1, CTR1, EIN2*). Data reported in **Table 2** show that the drought stress repressed the expression of the *nifH* gene, as already seen for the enzymatic activity, and that the down-regulation was stronger in *Ms*-1021 plants. Instead, a positive effect was observed for the expression of the *MtP5CS2* gene, whose transcript is known to accumulate in plants in response to osmotic stress. For *Ms*-RD64 plants an increased expression of this gene was measured after both stress treatments, whereas, for *Ms*-1021 plants a significant up-regulation was observed only under hydroponic condition.

**Table 2:** qPCR analysis of the nitrogenase-coding gene and those involved in plant response to abiotic stress.

Gene	Relative level^a^			
	Water deprivation		PEG-treatment	
	*Ms*-1021	*Ms*-RD64	*Ms*-1021	*Ms*-RD64
*nifH*	0.12 ± 0.05	0.56 ± 0.07	0.52 ± 0.14	0.83 ± 0.09
*MtP5CS2*	1.14 ± 0.05	1.34 ± 0.09	1.55 ± 0.22	1.71 ± 0.26
*MtAAO3*	1.52 ± 0.14	0.35 ± 0.04	2.26 ± 0.39	0.25 ± 0.04
*Mt-2g006330*	1.81 ± 0.27	1.04 ± 0.13	2.14 ± 0.31	1.05 ± 0.16
*Mt-8g095330*	1.48 ± 0.05	0.82 ± 0.09	1.50 ± 0.07	1.17 ± 0.17
*ETR1*	1.43 ± 0.21	0.95 ± 0.16	n.d.	n.d.
*CTR1*	1.44 ± 0.17	2.28 ± 0.11	n.d.	n.d.
*EIN2*	2.22 ± 0.20	1.29 ± 0.08	n.d.	n.d.

*Drought stress conditions were applied following the procedure described in the legend of **Table 1**. *^a^*Relative gene expression levels of selected genes from comparative CT method; 2^-ΔΔ*C*_*T*_^ > 1, genes more highly expressed in stressed-plants; 2^-ΔΔ*C*_*T*_^ < 1, genes more highly expressed in control plants. Values are the means ± SD of four different biological replicates. n.d. – Not determined*.

The *MtAAO3* gene, involved in the biosynthesis of ABA hormone, was significantly induced in *Ms*-1021-stressed plants and strongly repressed in *Ms*-RD64-stressed plants.

qRT-PCR analysis was also used to evaluate the expression level of two members of the sucrose non-fermenting related kinase subfamily 2 (SnRK2), the main activators of ABA signaling pathway, playing crucial roles in controlling cellular and physiological responses to abiotic stress in plant. For *Ms*-RD64-stressed plants no significant difference was detected for both genes under the two applied stress conditions. On the contrary, significant up-regulation of gene expression was observed for *Ms*-1021-stressed plants. The triggering effect was stronger for the *Mt-2g006330* gene.

Finally, two different responses were observed when measuring the expression of the main ethylene signaling genes connected with the defense responses in plant. The expression of the ETR1 receptor was unchanged in *Ms*-RD64-stressed plants and slightly induced in *Ms*-1021-stressed plants. The expression of the negative regulator CTR1 was slightly induced in *Ms*-1021-stressed plants and significantly up-regulated in *Ms*-RD64-stressed plants. An opposite result was registered for the expression of the EIN2 gene, a central component of the ethylene signaling: this gene was significantly induced in *Ms*-1021-stressed plants and only slightly induced in *Ms*-RD64-stressed plants.

### Ethylene Production

The endogenous ethylene (C_2_H_4_) emission promoted by controlled osmotic drought stress was measured from intact whole alfalfa plants subjected to PEG-treatment and compared with that of non-stressed control plants. The treatment with 15% PEG induced the production of very low ethylene levels for both *Ms*-1021 and *Ms*-RD64 plants (data not shown). By contrast appreciable ethylene levels were measured at the end of treatment with 20% (w/v) PEG-6000.

Up to 40% reduction in ethylene evolution was registered for *Ms*-RD64-stressed plants (57 ± 14 nmol C_2_H_4_/g FW/h) when compared to *Ms*-1021-stressed ones (94 ± 20 nmol C_2_H_4_/g FW/h).

Ethylene production by non-stressed control plants was very low and almost below the sensitivity limit of the instrument.

### Effect of Drought Stress on Soluble Sugars, Amino Acids, and Organic Acids

Targeted metabolic analysis was carried out on control and drought-stressed plants and led to the quantitative measurement of six metabolites (sucrose, glucose, malic acid, citric acid, glutamic acid, and proline) and the evaluation of the relative content for one metabolite (pinitol). The quantification was normalized to the dry weight of samples. The metabolic pattern obtained was different in shoots and roots (**Tables 3**, **4**). Although the proline accumulation was observed for the roots of both stressed samples, the increase measured for *Ms*-RD64-stressed plants was about twice than the one recorded for *Ms*-1021-stressed plants. A significant decrease in the citric acid level was observed only for *Ms*-RD64-stressed plants, whereas a lower level of glutamic acid was measured only for *Ms*-1021-stressed plants. In shoots (**Table 4**), sucrose and glutamic acid did not show significant alterations. The well-known osmoprotectants proline and glucose accumulated in both stressed samples with a stronger build-up observed for *Ms*-RD64-stressed plants. For the malic and citric acids, two intermediates of the tricarboxylic acid cycle (TCA), the following pattern has been observed: in *Ms*-RD64-stressed plants the citric acid, involved in the decarboxylating part of TCA, was greatly reduced, whereas the malic acid, a component of the non-decarboxylating part of TCA involved in a number of different cellular response to drought (Lehmann et al., 2012; Barchet et al., 2013), was significantly increased; in *Ms*-1021-stressed plants the citric acid level was not altered and the malic acid was reduced.

**Table 3:** Content of *root* metabolites for *Medicago* plants subjected to controlled osmotic drought stress (PEG-treatment).

Metabolite	*Ms*-1021 (mg/g DW)				*Ms*-RD64 (mg/g DW)			
	Control	PEG-treatment^a^	Ratio^b^	*P* value	Control	PEG-treatment^a^	Ratio^b^	*P* value
Sucrose	46.321 ± 2.127	35.161 ± 10.548	0.76	0.09	72.757 ± 14.094	56.535 ± 16.647	0.78	0.27
Glucose	26.647 ± 2.826	15.629 ± 3.397	0.59	<0.05	18.741 ± 4.895	11.305 ± 1.832	0.60	0.07
Malic acid	8.139 ± 0.947	7.490 ± 2.260	0.92	0.67	6.988 ± 0.86	9.600 ± 2.099	1.37	0.11
Citric acid	4.728 ± 0.424	4.965 ± 1.067	1.05	0.74	4.281 ± 0.346	2.639 ± 0.476	0.62	<0.05
Glutamic acid	2.177 ± 0.291	1.467 ± 0.297	0.67	<0.05	3.205 ± 0.923	2.233 ± 0.761	0.70	0.23
Proline	0.531 ± 0.026	1.092 ± 0.281	2.06	<0.05	0.514 ± 0.185	2.413 ± 0.441	4.69	<0.05

^a^*Half of the 28-day-old plants were transferred to the hydroponic water solution supplemented with 15% PEG-6000 for 28 h, while another half was kept in regular hydroponic solution until harvesting. **^b^**Ratio calculation was based on control condition. Data presented are the mean ± SD (*n* = 3). Data significance was evaluated by Student’s *t*-test*.

**Table 4:** Content of *shoot* metabolites for *Medicago* plants subjected to controlled osmotic drought stress (PEG-treatment).

Metabolite	*Ms*-1021 (mg/g DW)				*Ms*-RD64 (mg/g DW)			
	Control	PEG-treatment^a^	Ratio^b^	*P* value	Control	PEG-treatment^a^	Ratio^b^	*P* value
Sucrose	13.870 ± 3601	9.999 ± 1.597	0.72	0.16	7.146 ± 1748	6.604 ± 0.342	0.92	0.62
Glucose	5.353 ± 1131	12.469 ± 1.338	2.33	<0.05	4.904 ± 239	11.702 ± 0.95	2.39	<0.05
Malic acid	21.909 ± 1722	14.224 ± 2.735	0.65	<0.05	14.219 ± 1663	27.946 ± 3.834	1.97	<0.05
Citric acid	1.542 ± 583	1.558 ± 0.186	1.01	1.0	4.499 ± 1665	0.822 ± 0.184	0.18	<0.05
Glutamic acid	1.846 ± 206	1.734 ± 0.129	0.94	0.47	1.806 ± 936	1.352 ± 0.217	0.75	0.46
Proline	0.161 ± 0.024	0.295 ± 0.014	1.83	<0.05	0.120 ± 14	0.251 ± 0.076	2.09	<0.05

^a^*Half of the 28-day-old plants were transferred to the hydroponic water solution supplemented with 15% PEG-6000 for 28 h, while another half was kept in regular hydroponic solution until harvesting. **^b^**Ratio calculation was based on control condition. Data presented are the mean ± SD (*n* = 3). Data significance was evaluated by Student’s *t*-test*.

For the soluble sugar pinitol the ratio calculation based on control conditions showed that it was consistently accumulated in the shoots of *Ms*-RD64-stressed plants (Ratio = 3.99, *P* < 0.05) as compared to *Ms*-1021-stressed ones (Ratio = 1.31, *P* < 0.05). No statistically significant increase was observed in the roots of both *Ms*-1021- (Ratio = 1.09, *P* = 0.68) and *Ms*-RD64-stressed plants (Ratio = 2.72, *P* = 0.17).

## Discussion

The molecular and physiological mechanisms underlying drought stress in legume plants have been extensively studied but not completely clarified yet. Various molecular networks, including signal transduction, are involved in stress response (Chinnusamy et al., 2004; Huang et al., 2012).

Several drought-inducible genes have been studied including genes coding for enzymes required for the biosynthesis of various osmoprotectants, chaperones, and detoxification enzymes (Zhang et al., 2014).

In *Ms*-RD64-stressed plant cells the accumulation of osmoprotectants such as pinitol and proline, known to stimulate the activity of antioxidant enzymes, to protect the enzymatic systems, and to maintain water balance in alfalfa plants (Zhang et al., 2014), may have contributed to mitigating the negative effects of free radicals, thus minimizing the injury of oxidative stress triggered by drought.

Under drought stress conditions plant N availability is negatively affected as the nitrogenase activity is strongly reduced. Furthermore, the deleterious effects of drought on alfalfa performance are heavily targeted toward photosynthesis. The damages are caused by the inhibition of Rubisco activity, partly due to the lower Rubisco availability, and the impairment of thylakoid membrane activity, through inhibition of membrane-associated carriers and enzymes.

The *Ms*-RD64 plants had greater Rubisco availability that, under drought stress conditions, could be exploited to target a larger amount of N resources into the synthesis of osmoregulants such as proline. The increased production of nitrogenous compounds observed for the *Ms*-RD64-stressed plants did not lead to a feedback inhibition effect on nitrogenase activity as previously proposed by other authors (Aranjuelo et al., 2011, 2013).

In addition, the increased levels of NPQ measured for *Ms*-RD64 plants could have contributed to dissipating light energy and to lowering the efficiency of photosynthesis photochemical reactions, thus protecting them from drought stress exposure. Consistent with this, the levels of three thylakoid proteins, PsbO, PetC, and ATPase-B, were markedly increased or unaffected, respectively, in these plants.

In most cases, plants respond to environmental stresses by changing the level of endogenous phytohormones. ABA is identified as a stress hormone as it performs various functions under environmental stress conditions, particularly drought. Genetic modifications of many key enzymes involved in ABA biosynthesis have been performed in order to improve abiotic stress tolerance. Other phytohormones such as auxin, cytokinin, ethylene, brassinosteroids, jasmonates, and salicylic acid have also been suggested to be involved in response to abiotic stress. After water deprivation or PEG-treatment, the expression of the genes involved in the last step of the ABA biosynthesis and in the ABA-signaling was greatly reduced or unaffected, respectively, in *Ms*-RD64 plants as compared to *Ms*-1021 ones. This result led us to hypothesize that the leaf senescence delay and the protection of both nitrogen-fixing and photosynthetic functions observed for *Ms*-RD64 plants was not ABA-mediated. The up-regulation of the gene coding for the negative regulator of the ethylene signaling pathway, and the lower amount of ethylene produced by *Ms*-RD64-stressed plants might have led to changes in hormonal balance, thus relieving stress suffering by efficient activation of the main stress defense mechanisms.

Adaptation to drought also involves developmental changes, such as expanding the root system to maximize soil water capture. Transgenic poplar and potato plants, overexpressing the *Arabidopsis* YUCCA6 gene, exhibit auxin-overproduction phenotypes, such as increased root hair formation, and enhanced tolerance to drought stress (Kim et al., 2013; Ke et al., 2015).

Perennial ryegrass plants have already shown similar effects resulting from the alteration in endogenous hormone levels when steroid hormones, such as brassinosteroids, were exogenously applied to grass seedlings before salt stress (Wu et al., 2017).

The polar auxin transport pathway has also been involved in the regulation of the response to water stress in plants (Zhang et al., 2012).

It has been verified that the local delivery of IAA inside root nodules of *Ms*-RD64 plants altered root IAA polar transport and induced the development of more branched root systems with more secondary roots and nodules (Pii et al., 2006). The ability of the IAA-overproducing RD64 strain to induce rooting was also demonstrated in biotinization experiments when applied together with Carrizo citrange microcuttings into alginate matrix (Germanà et al., 2015).

The presence of more lateral roots in *Ms*-RD64 plants could be an adaptive strategy to increase water uptake under drought stress conditions by providing more adsorption surface.

The results reported in this study, combined with previous observations (Pii et al., 2006; Bianco and Defez, 2010a; Bianco et al., 2014), suggest that the better response to drought stress observed for *Ms*-RD64 plants was related to their phenotypic characteristics, mainly at the root level, and their better performances, mainly at the level of nitrogen and carbon metabolism.

The findings here reported highlight the sophisticated interplay between different phytohormones and their effects on plant growth and response to abiotic stresses. They also show how the plant microbiome plays a central role in the modulation of such phytohormones interplay.

The ectopic use of microbes to modulate the plant response to environmental changes is a remarkable advantage and could allow the selection of microbial consortia, helping plants to cope with different abiotic stresses.

## Author Contributions

RD contributed to the planning of the experiments, discussion of the results, and writing of the manuscript. AA performed most of the experiments. MD and AC performed metabolomic analyses. PP and LT performed photosynthesis-related experiments. CB wrote the manuscript and led the project.

## Conflict of Interest Statement

The authors declare that the research was conducted in the absence of any commercial or financial relationships that could be construed as a potential conflict of interest.

## Footnotes

1 http://www.walz.com/

2 http://www.agrisera.com/en/artiklar/plantalgal-cell-biology/index.html

3 http://imagej.nih.gov/ij/index.html
